# Reply to Jin, H. Comment on “Yoon et al. Heterogeneous Colorectal Cancer Risk in Women with Metabolic Dysfunction-Associated Steatotic Liver Disease by Age, Lipid, and Waist-Circumference: A Nationwide Cohort Study. *Cancers* 2026, *18*, 125”

**DOI:** 10.3390/cancers18152485

**Published:** 2026-08-03

**Authors:** Chang Ik Yoon, Hye Sun Lee, Soyoung Jeon, Jin Ah Lee, Dooreh Kim, Jong Min Lee

**Affiliations:** 1 Division of Breast Surgery, Department of Surgery, Seoul St Mary’s Hospital, College of Medicine, The Catholic University of Korea, Seoul 06591, Republic of Korea; fayn03@catholic.ac.kr (C.I.Y.); rlaenfpd@gmail.com (D.K.); 2 Biostatistics Collaboration Unit, Yonsei University College of Medicine, Seoul 03722, Republic of Korea; hslee1@yuhs.ac (H.S.L.); jsy0331@yuhs.ac (S.J.); 3 Department of Surgery, Yongin Severance Hospital, Yonsei University College of Medicine, Yongin 16995, Republic of Korea

We thank Jin [1] for their thoughtful commentary on our study [2]. The methodological points raised are valuable, and we appreciate the opportunity they provide for further discussion. We would, however, like to clarify our analytical rationale and offer our perspective on each of the concerns raised.

## 1. BMI-Dependent Misclassification of the Hepatic Steatosis Index

Jin raises the concern that the linear incorporation of body mass index (BMI) into the Hepatic Steatosis Index (HSI) may produce differential misclassification across body size strata, potentially enriching the “lean MASLD” subgroup with individuals harboring inflammatory liver conditions, and suggests that redefining MASLD using the Fatty Liver Index (FLI) would mitigate this concern. We appreciate this consideration, but several lines of evidence indicate that substituting the FLI for the HSI would not have improved the analytical integrity of our study.

First, the HSI and FLI demonstrate essentially equivalent discriminative performance for MASLD in East Asian populations. In a Japanese health-checkup cohort (*n* = 627), the AUROCs of the FLI and HSI for identifying MASLD were 0.90 and 0.88, respectively, with no statistically significant difference in DeLong testing; the equivalence was held in sex-stratified analyses (FLI vs. HSI: 0.87 vs. 0.86 in males, 0.93 vs. 0.90 in females) [3]. A recent validation study on youth using both NHANES and real-world clinical data similarly reported overlapping AUROCs (0.91 vs. 0.90 in NHANES; 0.93 vs. 0.93 in the clinical cohort) [4].

Second, the FLI exhibits substantial cutoff instability across populations, which is itself a major source of misclassification. In an asymptomatic Korean cohort of 4009 adults, the conventional Western FLI threshold of ≥60 yielded an AUROC of only 0.63 and required recalibration to FLI ≥ 29 to reach an AUROC of 0.82, with sex-specific optimal cutoffs as low as 18 in women [5]. Comparable recalibration has been reported in Japanese (cutoff 12 in women, 39 in men) [3] and pediatric populations [4]. No FLI cutoff has been prospectively validated in a Korean middle-aged female population comparable to ours, whereas the HSI threshold of ≥36 was originally derived from a Korean cohort and remains directly applicable to our study population [6].

Third, and most consequentially, the FLI would have introduced collinearity with the variables we examined as effect modifiers. The FLI formula incorporates BMI, waist circumference, triglycerides, and gamma-glutamyl transferase as direct components, whereas HSI relies only on the AST/ALT ratio, BMI, sex, and diabetes. Because waist circumference and dyslipidemia (driven largely by triglyceride elevation) were two of our three pre-specified subgroup variables, defining MASLD by the FLI would have meant stratifying on variables that were themselves embedded in the exposure definition. In this respect, the choice of the HSI was not merely operational but methodologically necessary to preserve the validity of our effect modification analyses.

Finally, the commentator’s underlying biological hypothesis—lean women classified as having MASLD by the HSI may harbor occult hepatic inflammation—is, in our view, not contradictory to our findings. The growing literature on lean MASLD demonstrates that non-obese individuals with hepatic steatosis show disproportionately high rates of insulin resistance, visceral adiposity relative to BMI, and systemic low-grade inflammation [7,8], all of which are themselves established drivers of colorectal carcinogenesis through the insulin–IGF axis and chronic inflammatory pathways [9]. A recent propensity score-matched analysis using the TriNetX network, presented at the 2026 ASCO Gastrointestinal Cancers Symposium, further demonstrated that lean MASLD was associated with a significantly elevated CRC risk compared with non-lean MASLD (HR 1.53; 95% CI 1.13–2.06; *p* = 0.006), independent of HSI-based classification [10]. Whether the lean MASLD phenotype reflects steatosis alone or steatosis coexisting with subclinical inflammation, the resulting elevation in CRC risk constitutes a biologically meaningful signal. We would also emphasize that our MASLD definition required at least one metabolic abnormality in addition to HSI ≥ 36, in accordance with current international consensus criteria.

## 2. Statin-Related Indication Bias

We appreciate Jin’s astute observation regarding the potential confounding effect of statin use on the MASLD–CRC association in women with dyslipidemia. We agree that this is a plausible and likely contributor to the null finding observed in the dyslipidemic subgroup. Indeed, we addressed this mechanism in our original Discussion, noting that statin therapy inhibits HMG-CoA reductase, lowers secondary bile acid production, and may attenuate colorectal carcinogenesis. Large-scale meta-analyses including 42 studies have reported that statin use is associated with a modest but significant reduction in CRC risk (relative risk: 0.90; 95% CI: 0.86–0.95) [11], and a recent Korean nationwide cohort study confirmed this chemopreventive potential specifically among dyslipidemic patients [12]. We therefore concur with the commentator that the absence of an MASLD–CRC association in dyslipidemic women may reflect, at least in part, pharmacological masking via statin therapy rather than a true biological absence of risk.

However, we would like to draw attention to what we believe is the more clinically consequential implication of our findings. Regardless of whether the null result in the dyslipidemic subgroup is attributable to statin chemoprevention or to other mechanisms, women with dyslipidemia remain a recognized higher-risk group for CRC and are typically already engaged with the healthcare system through lipid management and cardiovascular risk surveillance. The clinically actionable signal from our study, lies in the opposite stratum: normolipidemic women with MASLD, who appear metabolically healthy by conventional lipid criteria, are not receiving statin therapy and may not be flagged for intensified cancer surveillance—yet, in our cohort, they demonstrated a significantly elevated CRC risk (HR: 1.15; 95% CI: 1.03–1.28). This subgroup represents a population in whom MASLD may serve as an otherwise unrecognized marker of carcinogenic risk, precisely because the protective umbrella of statin therapy and clinical attention is absent. The take-home message we wish to convey is not that dyslipidemia is protective, but that the absence of dyslipidemia should not be interpreted as the absence of metabolic CRC risk when MASLD is present.

## 3. Unmeasured Confounding

We thank the commentator for raising this important point. Unmeasured confounding is an inherent limitation of all retrospective, claim-based epidemiological studies, and we fully acknowledge that family history of CRC and aspirin/NSAID use—along with other potentially relevant variables such as dietary patterns, gut microbiome composition, and post-baseline medication changes—could not be incorporated into our analysis. We recognized these limitations in the Discussion section of our original manuscript and agree that they warrant cautious interpretation of the observed associations. We are grateful to Jin for reinforcing the importance of these considerations, which we believe should be addressed in future prospective studies with richer covariate data.

## 4. Concluding Remarks

We thank Jin for constructive engagement with our work. While we acknowledge the methodological limitations discussed above, we believe the core findings of our study—that MASLD is independently associated with CRC risk in Korean women, particularly among those who are younger, normolipidemic, or non-obese—remain clinically meaningful and warrant attention. We hope this exchange will encourage further research to validate and extend these observations in diverse populations.

## Data Availability

No new data were created or analyzed in this study. Data sharing is not applicable to this article.
